# Inhibitors of HSP90 in melanoma

**DOI:** 10.1007/s10495-019-01577-1

**Published:** 2019-10-28

**Authors:** Aleksandra Mielczarek-Lewandowska, Mariusz L. Hartman, Malgorzata Czyz

**Affiliations:** Department of Molecular Biology of Cancer, Medical University of Lodz, 6/8 Mazowiecka Street, 92-215 Lodz, Poland

**Keywords:** Apoptosis, Chaperone, HSP70, HSP90 inhibitors, Melanoma, Targeted therapy

## Abstract

HSP90 (heat shock protein 90) is an ATP-dependent molecular chaperone involved in a proper folding and maturation of hundreds of proteins. HSP90 is abundantly expressed in cancer, including melanoma. HSP90 client proteins are the key oncoproteins of several signaling pathways controlling melanoma development, progression and response to therapy. A number of natural and synthetic compounds of different chemical structures and binding sites within HSP90 have been identified as selective HSP90 inhibitors. The majority of HSP90-targeting agents affect N-terminal ATPase activity of HSP90. In contrast to N-terminal inhibitors, agents interacting with the middle and C-terminal domains of HSP90 do not induce HSP70-dependent cytoprotective response. Several inhibitors of HSP90 were tested against melanoma in pre-clinical studies and clinical trials, providing evidence that these agents can be considered either as single or complementary therapeutic strategy. This review summarizes current knowledge on the role of HSP90 protein in cancer with focus on melanoma, and provides an overview of structurally different HSP90 inhibitors that are considered as potential therapeutics for melanoma treatment.

## Introduction

The number of patients diagnosed with melanoma increases every year. In 2019, over 90,000 of new melanoma cases have been estimated to be diagnosed in United States alone [1]. Environmental factors, especially UV exposure, familiar history and genetic factors are amongst major causes of melanoma [2–4]. The most common mutations are found in genes encoding components of the RAS/RAF/MEK/ERK (MAPK) signaling pathway, and they lead to a constitutive activity of this cascade [5]. Algorithms for current treatment of melanoma patients include vemurafenib, dabrafenib and encorafenib, targeting mutated BRAF (B-RAF proto-oncogene, serine/threonine kinase); trametinib, cobimetinib and binimetinib that inhibit the activity of MEK1/2 (mitogen-activated protein kinase kinase), as well as immune checkpoint inhibitors including nivolumab and pembrolizumab binding to PD-1 (programmed cell death protein 1) and ipilimumab inhibiting CTLA-4 (cytotoxic T-lymphocyte antigen 4) [6–9]. However, available therapies have several limitations. Melanoma cells develop resistance towards BRAF and MEK inhibitors through a number of genetic and epigenetic mechanisms [10–13]. Resistance emerges through upregulation of expression of mutated *BRAF*, alternative splicing of BRAF transcript, secondary *BRAF* mutations, mutations in genes encoding MEK1/2 and RAS, reactivation of COT (cancer osaka thyroid oncogene) activity, dimerization of CRAF (RAF-1 proto-oncogene, serine/threonine kinase), which all can lead to the hyperactivation or recovery of the MAPK pathway activity. In addition, a loss of functional PTEN (phosphatase and tensin homolog) and enhanced activity of the PI3K (phosphatidylinositol 3-kinase)/AKT/mTOR (mechanistic target of rapamycin kinase) pathway, a suppression of BIM (BCL-2 interacting mediator of cell death), a loss of STAG2 (stromal antigen 2) or STAG3 (stromal antigen 3) that are the subunits of cohesion complex, an increase in cyclin D1 level, enhanced expression of several microRNAs, and expression of resistance-associated genes including *AXL*, *PDGFRB* (platelet-derived growth factor receptor beta) and *EGFR* (epidermal growth factor receptor) have been demonstrated to contribute to resistance of melanoma cells. In addition to cell-intrinsic mechanisms, growth factors derived from stromal cells and hypoxia can modulate melanoma cell sensitivity to targeted drugs [10–12, 14–16], and long-term therapy with BRAF^V600E^ inhibitor can develop resistance to other drugs including dacarbazine [17]. Resistance to immunotherapy can also emerge, and melanoma cells resistant to PD-1 inhibitors show upregulation of receptors VISTA (V-domain Ig suppressor of T cell activation) and TIM-3 (T-cell immunoglobulin and mucin domain-containing 3), as well as acquisition of mutations in genes encoding JAK1 (Janus kinase 1), JAK2 (Janus kinase 2) and B2 M (beta-2-microglobulin), which results in reduced sensitivity to T-cell mediated killing [18, 19]. Therefore, there is still an urgent need for alternative therapeutic approaches for melanoma treatment, and new targets need to be identified.

HSP90 (heat shock protein 90) that is one of the crucial mediators of cellular physiology [20], is also recognized as a key facilitator of cancer cell survival [21]. This review delineates recent advances in our understanding of HSP90 function in melanoma cells and provides information on HSP90 inhibitors as potential drugs for melanoma treatment.

### HSP90: structure and regulation of activity

**Fig. 1:**
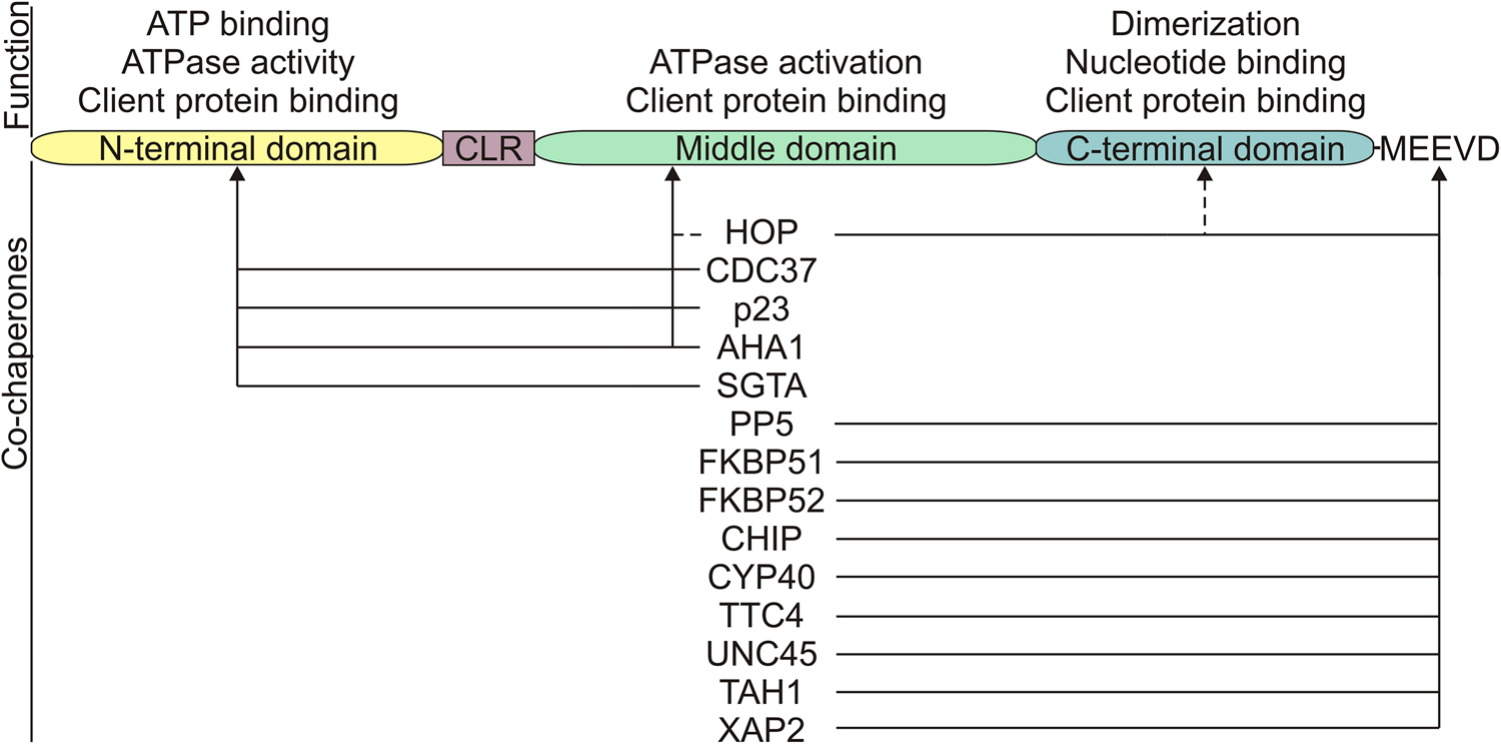
Schematic representation of HSP90 protein domain structure. Functions of each domain and HSP90-interacting co-chaperones with their binding sites are shown. Dashed lines represent alternative binding sites. AHA1: activator of HSP90 ATPase protein 1; ATP: adenosine triphosphate; CDC37: cell division cycle 37; CHIP: carboxyl terminus of HSP70-interacting protein; CLR: charged linker region; CYP40: cyclophilin 40; FKBP51: FK506-binding protein 5; FKBP52: FK506-binding protein 4; HOP: homeodomain-only protein; PP5: protein phosphatase 5; SGTA: small glutamine rich tetratricopeptide repeat-containing alpha; TAH1: telomere-associated homeobox-containing protein 1; TTC4: tetratricopeptide repeat domain 4; UNC45: smooth muscle cell-associated protein 1; XAP2: HBV X-associated protein 2

The HSP90 family of proteins includes the cytosolic HSP90α, HSP90β and HSP90 N isoforms, ER (endoplasmic reticulum)-residing member GRP94 (glucose-regulated protein 94) and mitochondrial protein TRAP1 (tumor necrosis factor receptor-associated protein 1) [22]. HSP90β is constitutively expressed, whereas HSP90α is induced in response to stress [23]. HSP90 homologues share conserved domains including an N-terminal domain (NTD; ~ 25 kDa), a middle-domain (MD; ~ 35 kDa), a C-terminal domain (CTD; ~ 10 kDa) and a flexible charged linker between NTD and MD [24–26]. In addition, HSP90α and HSP90β possess C-terminal Met-Glu-Glu-Val-Asp (MEEVD) motif [22]. HSP90 domains play specific roles (Fig. 1). NTD predominantly exerts ATPase activity, MD assists in activation of ATPase activity, whereas CTD is responsible for HSP90 dimerization, which is essential for its chaperone function [22, 27]. In addition, HSP90 domains serve as binding sites for the client proteins and co-chaperones (Fig. 1).

**Fig. 2:**
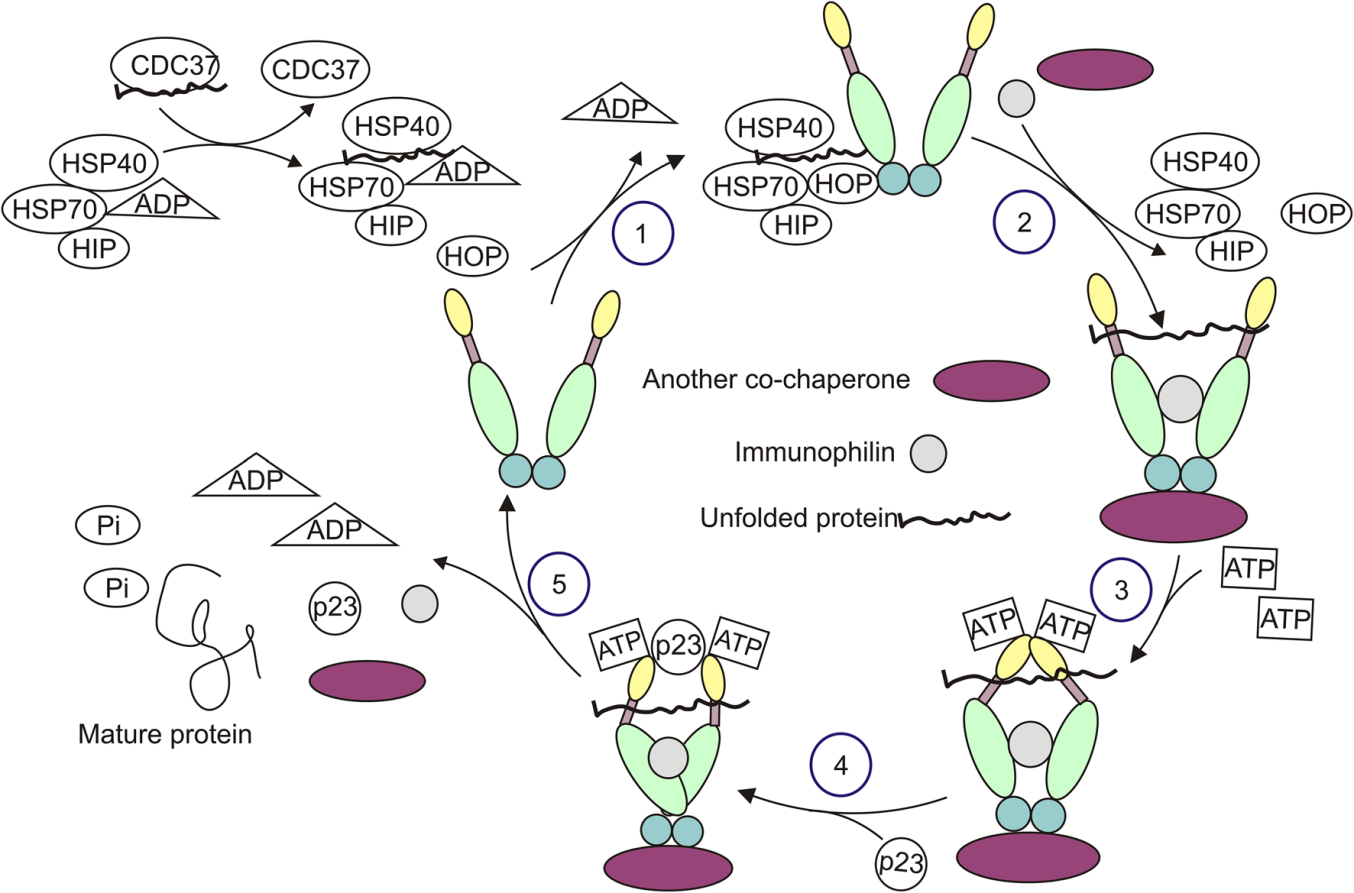
Exemplary chaperone cycle of HSP90. The consecutive steps are marked with numbers. Unfolded client protein of HSP90 is transferred from CDC37 to HSP70/HSP40/HIP/ADP complex, and becomes attached to HSP90 in an open conformation with assistance of HOP (1). Then, other co-chaperones and immunophilins are bound to the HSP90 homodimer, while HSP40, HSP70, HIP and HOP being released (2). Binding of ATP to the N-terminal domain of HSP90 switches the protein from an open to close conformation (3). Subsequently, p23 is attached (4), which is followed by ATP hydrolysis, and the release of mature protein, co-chaperones and immunophilins as well as conformational change of HSP90 (5). HSP40: heat shock protein 40; HIP: Hsc70-interacting protein

HSP90 contributes to folding and remodeling of proteins, assists in assembly of multi-protein complexes, and enables ligand binding to receptors [28]. HSP90 activity depends on the cooperation with co-chaperones and immunophilins, and is associated with highly dynamic conformational rearrangements during the chaperone cycle [26] (Fig. 2). In the absence of ATP, HSP90 predominantly adopts a V-shaped open conformation. ATP binding to NTD triggers closed state of HSP90, which is preceded by an intermediate steps involving the contribution of the N-terminal “lid” region. Closed state of HSP90 is crucial for ATP hydrolysis as it involves a reposition of a catalytic loop in the middle domain to activate ATPase activity in N-terminal domain. When HSP90 reaches the fully closed state, ATP undergoes hydrolysis which is followed by a disassembly of a multi-protein complex. ATPase cycle of HSP90 is symmetric as both ATP molecules are disrupted simultaneously. In addition, conformational cycle of HSP90 similarly appears in the presence and absence of the client proteins [28–30]. Co-chaperones substantially regulate HSP90 function as they diversely modulate HSP90 chaperone cycle and act as adaptors for specific client recruitment [22]. Regulation of HSP90 activity can be additionally modulated at the transcriptional level and through posttranscriptional modifications. Transcriptional regulation of *HSP90* expression is mainly controlled by HSFs (heat shock factors), which bind to HSE (heat shock element) located in the promoter region of *HSP90* [31, 32]. HDAC6 (histone deacetylase 6)-mediated regulation of HSP90 stability, posttranslational modifications of cytosolic HSP90 that include phosphorylation, acetylation, methylation, ubiquitylation and S-nitrosylation have been extensively discussed elsewhere [30, 33–35].

### HSP90 in melanoma

**Fig. 3:**
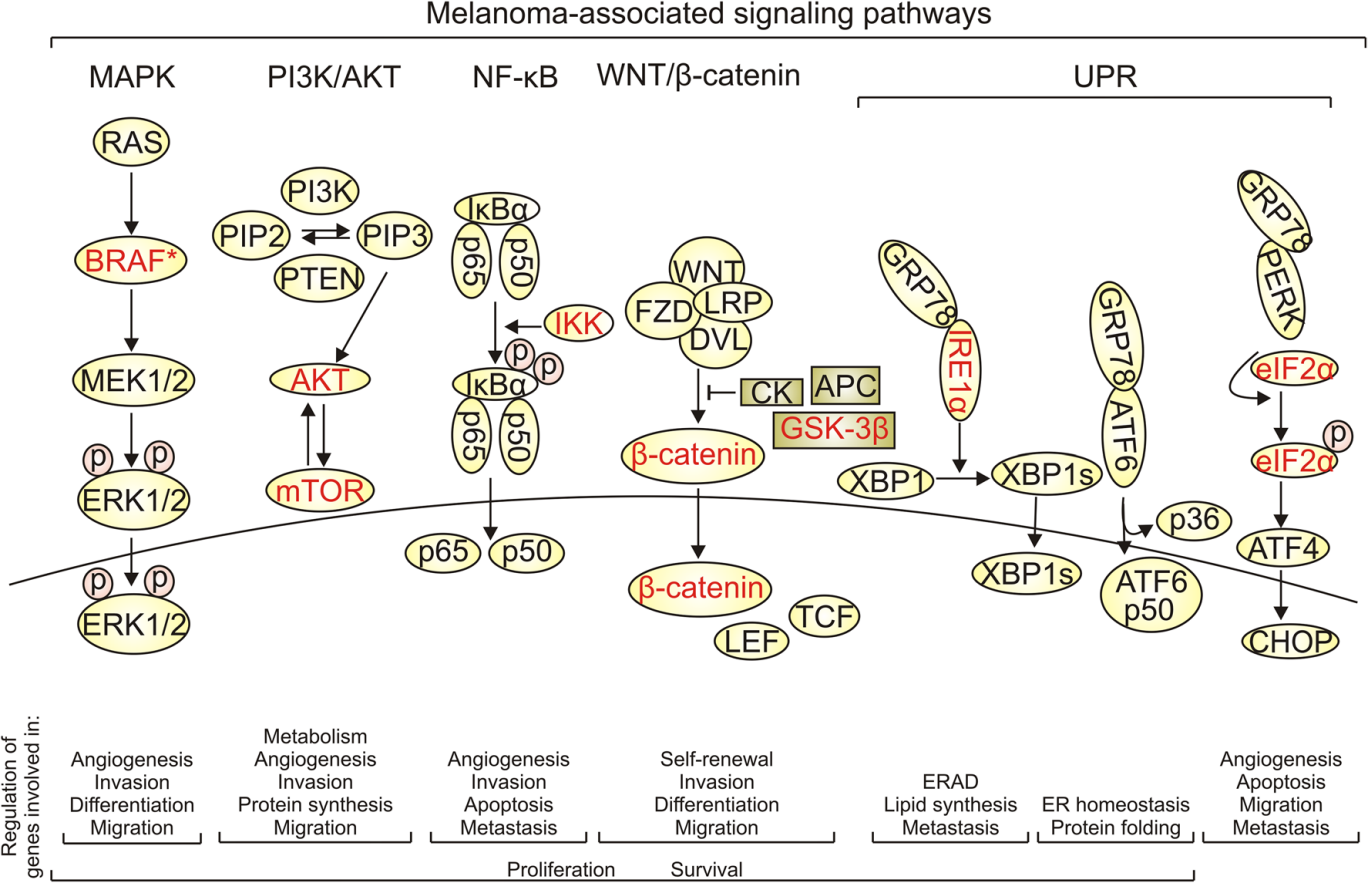
Major melanoma-associated signaling pathways, and their roles in melanoma. Proteins identified as direct HSP90 clients are depicted in red. *only BRAF mutants but not wild-type protein are reported as HSP90 clients in cutaneous melanoma. APC: adenomatous polyposis coli; CK: creatine kinase; DVL: dishevelled; ERAD: endoplasmic reticulum-associated protein degradation; FZD: frizzled; GSK-3β: glycogen synthase kinase 3 beta; IKK: IκB kinase; LEF: lymphoid enhancer-binding factor 1; LRP: low density lipoprotein receptor-related protein; PIP2: phosphatidylinositol biphosphate; PIP3: phosphatidylinositol(3,4,5)trisphosphate; TCF: T-cell factor

*HSP90* is usually overexpressed in cancer [28]. While HSP90 level is low in benign melanocytic nevi, it increases during melanoma progression [36]. Consequently, a high level of HSP90 was assessed in more than 50% of melanoma tumors [37, 38]. Although high *HSP90* expression is not a predictive factor for patient survival, HSP90 level significantly correlated with the Clark level and increased Breslow depth in primary melanomas [36]. Despite intracellular presence of HSP90, it is also identified on melanoma cell surface suggesting that HSP90 might be an immunorelevant target [39, 40]. It has been shown that a membrane-bound HSP90 facilitates immune clearance of dying cells [41]. In addition, HSP90 can be secreted into the extracellular space [42]. HSP90 was detected in serum of melanoma patients at significantly higher levels than in healthy controls [43]. As extracellular HSP90 can promote cell motility and angiogenesis [44], serum level of HSP90 might be considered as a putative biomarker of melanoma progression. HSP90 controls folding and maturation of more than 200 proteins, and up-to-date list of HSP90 clients and interactors is available at the Picard’s Lab website [https://www.picard.ch/downloads/Hsp90interactors.pdf]. HSP90 plays a multifactorial role in melanoma. HSP90 isoform was found in melanoma-derived exosomes and was considered as a part of ‘education’ program for bone marrow cells creating a pre-metastatic niche for melanoma cells [45]. Formation of a triple HSP90/HIF-1α/BCL-2 (B-cell CLL/lymphoma 2) complex results in stabilization of HIF-1α (hypoxia-inducible factor 1) under hypoxic conditions [46]. In addition, melanoma development and progression are substantially dependent on several key signaling pathways, all including essential oncoproteins identified as HSP90 client proteins (Fig. 3). MAPK signaling pathway is responsible for melanoma cell proliferation, differentiation, survival, invasion and angiogenesis [9, 47]. This signaling cascade is constitutively active in the majority of melanomas as a result of genetic alterations in *BRAF*, *RAS* or *NF1* (neurofibromin 1) [48–50]. Most frequent mutations are found in *BRAF* (40–60% of melanoma patients) and *NRAS* (15–20%). Mutations in *BRAF* are mainly associated with a substitution of valine in codon 600, and valine can be substituted with either glutamic acid or lysine in up to 90% and 10–20% of patients harboring mutation in *BRAF*, respectively [5, 48]. Interestingly, HSP90 is required for folding of a protein product of mutated *BRAF*, whereas wild-type BRAF is not stabilized by HSP90 in cutaneous melanoma cells [51, 52]. By increasing intracellular protein load, oncogenic MAPK signaling broadly affects UPR (unfolded protein response) pathways involved in cell fate decision during prolonged ER stress [53, 54]. It has been also demonstrated that sustained activity of IRE1α (inositol-requiring enzyme 1 alpha) and ATF6 (activating transcription factor 6) promotes adaptation of melanoma cells to proteotoxic stress [55], which supports cancer progression [56]. HSP90 also contributes to the regulation of PI3 K/AKT signaling, which is involved in melanoma cell proliferation, migration and survival [57, 58], and it is often activated in melanoma cells resistant to BRAF and MEK inhibitors [59]. HSP90 may also control cell metabolism and protein synthesis through a cross-talk between PI3 K/AKT cascade and mTOR [57, 60, 61] as both AKT and mTOR are HSP90 client proteins (Fig. 3). IKKs (IκB kinases), other HSP90 client proteins, control NF-κB (nuclear factor kappa B) activation [62]. NF-κB signaling pathway is constitutively active in melanoma cells, where it regulates expression of genes involved in apoptosis, cell cycle, invasion and angiogenesis [63]. Activation of NF-κB is also associated with emergence of resistance to BRAF inhibitors [64–66]. WNT (Wingless-type)/β-catenin signaling pathway takes part in melanoma development, cell self-renewal and migration [67, 68]. It has been also shown that ABCB5 (ATP-binding cassette, sub-family B, member 5) contributes to WNT-dependent expression of *CXCL8* (C-X-C motif chemokine ligand 8) encoding interleukin-8 to support a slow-cycling and chemoresistant phenotype of melanoma cells [69]. In addition, the enhanced activity of WNT signaling pathway was found in melanospheres [70]. AXL, another HSP90-interacting protein relevant for melanoma [71], has been identified as a driver of resistance to targeted drugs in melanoma [72], and a regulator of metastasis-promoting phenotype of melanoma cells [73].

### HSP90 inhibitors

**Fig. 4:**
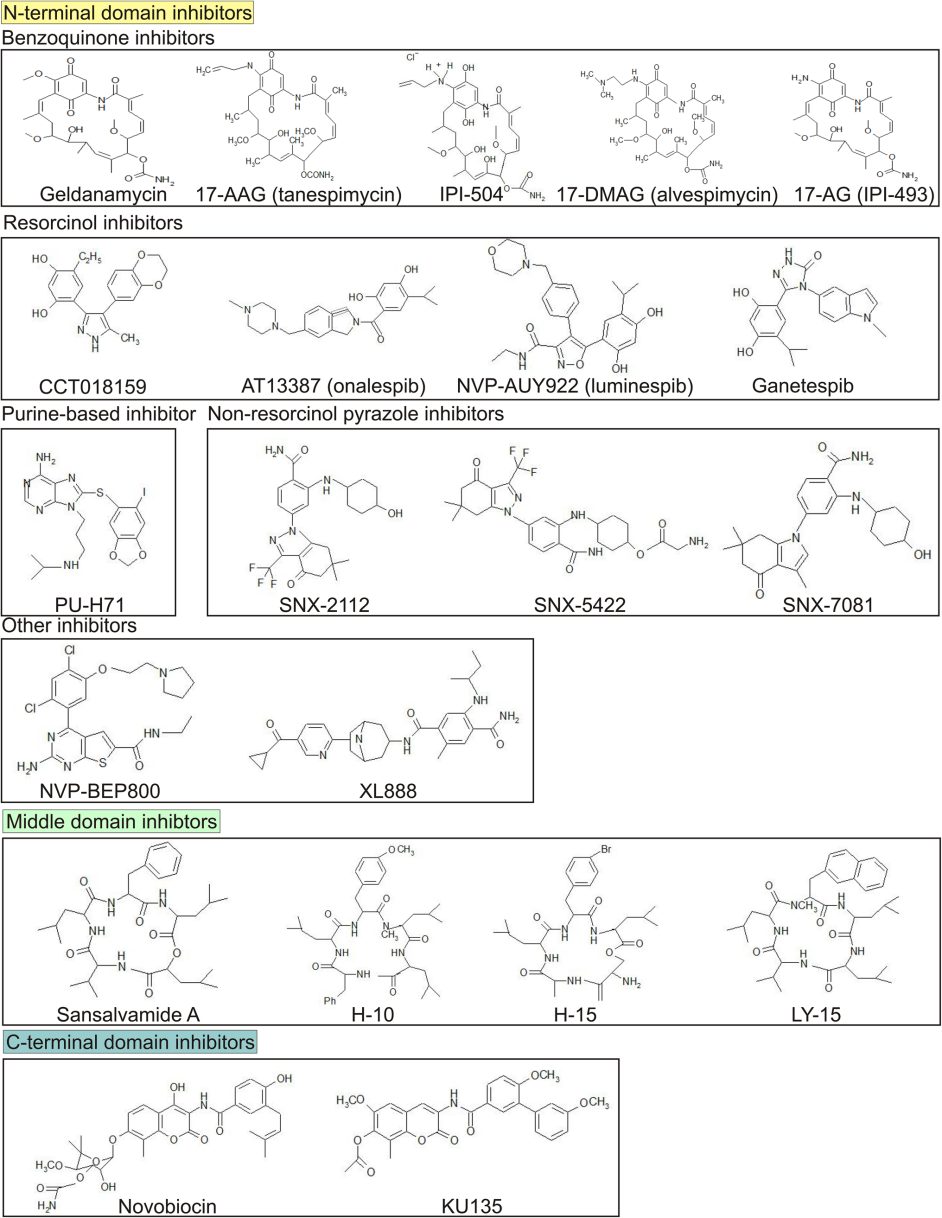
HSP90 inhibitors exerting anti-melanoma activity. Compounds were classified based on their binding sites and similarity in chemical structure

The role of HSP90 in melanoma development and progression makes this protein a promising therapeutic target. The rationale for targeting HSP90 is supported not only by a high level of this protein in cancer cells, but also by cancer cell-selective formation of HSP90 multi-chaperone complexes exerting a high ATPase activity [74]. In addition, HSP90 has been identified as a crucial regulator of melanoma cell phenotype, and inhibition of HSP90 has substantially affected both commercially available and primary melanoma cell lines [75], also those resistant to currently available therapeutics [76]. A number of natural and synthetic compounds of different chemical structures and binding sites have been identified as selective HSP90 inhibitors (Fig. 4). N-terminal domain inhibitors act by disrupting the interaction between ATP and ATP-binding pocket, and they restrain HSP90 in the ADP-bound state that leads to ubiquitylation and subsequent proteasomal degradation of the client proteins [77]. In turn, C-terminal domain inhibitors destabilize the chaperone complex and induce a release of co-chaperones and degradation of client proteins [78, 79]. Inhibitors of the middle domain of HSP90 directly or allosterically disrupt interactions between HSP90 and C-terminal binding proteins [80].

### N-Terminal domain inhibitors

#### Benzoquinone inhibitors

Geldanamycin is a benzoquinone, ansamycin antibiotic of natural origin isolated from *Streptomyces hygroscopicus*, and exerts a potent activity against different types of cancer cells by competing with ATP for binding to the N-terminal domain of HSP90 [81]. However, substantial hepatotoxicity and unsatisfactory solubility of geldanamycin [82, 83] enforced the research to develop geldanamycin derivatives. 11-methoxy-17-formyl-17-demethoxy-18-O-21-*O*-dihydrogeldanamycin was isolated from another strain of *Streptomyces hygroscopicus* (A070101), and exerted a marked cytotoxicity against cancer cells including melanoma [84]. In addition, several 17-substituted semi-synthetic derivatives of geldanamycin exerted a promising activity while being less toxic against normal cells. The methoxy substituent of benzoquinone moiety in geldanamycin was replaced by an allyloamine group (17-AAG; tanespimycin), dimethyl-aminoethylamine group (17-DMAG; alvespimycin) or changed for an amine group (17-AG, IPI-493) [81]. Moreover, 17-AAG hydrochloride (IPI-504) was obtained by a reduction of benzoquinone moiety in 17-AAG to hydrochinon [85].

Activity of ansamycin HSP90 inhibitors was broadly studied in melanoma cells. It was demonstrated that 17-AAG induced degradation of BRAF^V600E^ and other BRAF mutants, but not wild-type BRAF in cutaneous melanoma cells [51, 52]. However, HSP90 inhibition by 17-AAG or 17-DMAG affected wild-type BRAF in uveal melanoma cell lines [86]. Consequently, geldanamycin derivatives inactivated MAPK signaling that was shown as reduced level of phosphorylated ERK1/2 [86–89]. This effect was similarly observed in melanoma cells harboring mutation in *NRAS* [89, 90]. 17-DMAG also decreased levels of phospho-ERK1/2 and phospho-AKT under hyperthermic conditions [90]. As a result, activity of HSP90 inhibitors was associated with downregulation of cyclin D1 and inhibition of cell proliferation [86, 87]. Geldanamycin derivatives differed in a cytostatic potential as shown for 17-DMAG that was more effective at inhibiting melanoma cell proliferation than 17-AAG [90]. In addition, 17-AAG inhibited vemurafenib-mediated paradoxical activation of ERK1/2 [91]. It was also demonstrated that geldanamycin and its analogs induced cell death [89, 90, 92]. 17-AAG and 17-DMAG induced apoptosis associated with the activation of caspase-9, caspase-2 and caspase-7, and PARP (poly-ADP ribose polymerase) cleavage [90]. 17-AG was more potent in caspase-3/7 activation than geldanamycin in patient-derived melanoma cells [89], although geldanamycin exerted lower IC_50_ values for anti-clonogenic activity than 17-AG [93]. 17-AG-mediated apoptosis was associated with attenuation of cytoprotective IRE1α-XBP1s (spliced X-Box binding protein 1) axis in melanoma cells harboring either BRAF^V600E^ or NRAS^Q61R^ variant [89].

In addition to cytostatic and cytotoxic effects of ansamycin HSP90 inhibitors, these compound can affect melanoma cell phenotype. 17-AAG induced differentiation by increasing protein levels of tyrosinase and PMEL/gp100 (premelanosome protein/glycoprotein 100) in both *BRAF*-mutated and wild-type *BRAF* melanoma cell lines [87]. In another study, 17-AAG increased expression of *DCT* (dopachrome tautomerase) and *TYRP1* (tyrosinase-related protein 1) encoding pigmentation-related proteins, and elevated melanin production in melanoma cells [91]. Additionally, 17-AAG increased glycerophosphocholine levels which was coupled with an elevated content of cytoplasmic mobile lipid droplets and enhanced fatty acid signaling suggesting that HSP90 inhibition can also broadly affect metabolism of melanoma cells [87].

Geldanamycin-related HSP90 inhibitors upregulate co-chaperones and stress-related response genes that can affect cell sensitivity to these drugs. Geldanamycin, 17-AAG, 17-DMAG and 17-AG induced expression of *HSP70* (heat shock protein 70) [87, 89, 94–96], however, this effect was transient and silenced already after 22 h in melanoma cells exposed to 17-AG [89]. HSP70 upregulation can be the outcome of HSF-1 (heat shock factor 1) activation to induce expression of genes encoding heat shock proteins [97]. Accumulation of HSP70 reduced the extent of cell death induction in response to HSP90 inhibition [98]. In addition, 17-AG upregulated ER-located chaperone protein GRP78 (glucose-regulated protein 78) in a manner similar to the induction of HSP70 [89].

Cell sensitivity to ansamycin inhibitors of HSP90 was also associated with expression of *NQO1* encoding NAD(P)H:quinone oxidoreductase 1 that converted these compounds to hydroquinone to enhance their activity by increasing hydrogen bonding [99]. It was reported that NQO1^P187S^ variant exerted diminished activity compared with wild-type *NQO1* [99], but genetic alterations affecting a His^80^ residue in this protein could compensate for P187S substitution [100]. Loss of *NQO1* expression and acquisition of NQO1^P187S^ variant contributed to the development of resistance to 17-AAG [101]. Accordingly, sensitivity of melanoma cells to 17-AAG was related to *NQO1* overexpression [102]. In NQO1^low^ melanoma cells, combination of 17-AAG and cisplatin exerted cooperation, which was driven by cisplatin-mediated induction of reactive oxygen species and up-regulation of *NQO1* [102]. Interestingly, melanoma cells harboring P187S variant of NQO1 were susceptible to 17-AG-induced apoptosis, although the occurrence of cell death was delayed compared with melanoma cells harboring wild-type *NQO1* [89]. This could result from similar affinity of quinone and hydroquinone forms of 17-AG to HSP90 [103] as NQO1-independent cell response to 17-AG was also reported in another study [104].

While the molecular effects of 17-AAG in melanoma cells are diverse, no objective clinical response to this drug was reported in a phase II trial in patients with metastatic melanoma [105]. In addition, several adverse effects of grade 2 and 3 severity were reported, including nausea, vomiting and fatigue [105]. Geldanamycin derivatives cooperated with other drugs or therapeutic approaches. Combination of 17-AAG and tipifarnib, a farnesyltransferase inhibitor, was cooperatively cytotoxic against melanoma cell lines derived from advanced stage tumors, but not against cells from radial growth phase melanoma [106]. Drug combination induced apoptosis through mitochondrial pathway as evidenced by increase in caspase-3 and caspase-9 activation, and DNA fragmentation [106]. By degrading HIF-1α protein, 17-AAG synergistically cooperated with glucose analog (2-DG) and imiquimod (a ligand for Toll-like receptor 7/8) to induce cell apoptosis and inhibit melanoma tumor growth in vivo [107]. Combination of 17-AAG and PI3 K inhibitor diminished ERK1/2 and AKT phosphorylation, and exerted enhanced anti-melanoma activity than either drug used alone [88]. Geldanamycin [108] and 17-DMAG cooperated with hyperthermia to more potently inhibit melanoma cell proliferation and increase the number of apoptotic and necrotic cells in a time-dependent manner [90]. 17-AG enhanced activity of MAPK pathway inhibitors, vemurafenib and trametinib, in induction of apoptosis that might result from concurrent inhibition of IRE1α and ERK1/2 activities [89]. Interestingly, it was also demonstrated that prolonged treatment with 17-AAG could develop resistance to a spectrum of structurally distinct inhibitors of HSP90, and acquired resistance could be overcome by HDAC inhibitors [109].

### Resorcinol inhibitors

#### CCT018159

CCT018159 is a synthetic 3,4-diarylpyrazole resorcinol inhibitor of N-terminal ATPase activity of HSP90 [110]. Lack of a benzoquinone moiety in CCT018159 may determine lower hepatotoxicity than this observed for ansamycin inhibitors of HSP90 [111]. CCT018159 displayed a number of similar activities compared with geldanamycin and geldanamycin derivatives including induction of *HSP70* expression [111], depletion of melanoma-related oncoproteins such as BRAF^V600E^, CRAF, CDK4 (cyclin-dependent kinase 4), ERBB2 (receptor tyrosine protein kinase ERBB2) [111], attenuation of ERK1/2 activity [87, 111] and upregulation of genes involved in melanoma cell differentiation [87]. In addition, CCT018159 caused a substantial accumulation of melanoma cells in the G1 phase of cell cycle, and induced apoptosis [87, 111]. Melanoma cell response to CCT018159 was independent of *NQO1* expression and the level of P-glycoprotein/ABCB1 (ATP binding cassette subfamily B member 1) involved in drug efflux [111].

#### AT13387 (onalespib)

AT13387 is a long-acting inhibitor that interacts with the N-terminal ATPase catalytic site of HSP90. AT13387 exerted high activity against cancer cells addicted to several oncoproteins including receptor tyrosine kinases such as EGFR, ERBB2, c-MET (hepatocyte growth factor receptor) and FLT3 (Fms-related tyrosine kinase 3) [112]. AT13387 also depleted HSP90 client proteins including CRAF, BRAF^V600E^ and AKT in a concentration-dependent manner [112, 113] leading to attenuation of MAPK and AKT signaling pathways, also in a three-dimensional model of melanoma [113]. Notably, molecular effects of AT13387 activity were still visible 48 h after drug wash out [112]. AT13387 at low nanomolar concentration efficiently inhibited melanoma cell proliferation compared with other types of cancer cells [112], induced apoptosis and delayed tumor growth when used either alone or in combination with vemurafenib [113]. Notably, AT13387 delayed the emergence of resistance to vemurafenib in vitro and in vivo. In addition, melanoma cells resistant to vemurafenib or resistant to a combination of BRAF and MEK inhibitors were sensitive to AT13387 [113]. Similarly to other N-terminal HSP90 inhibitors, AT13387 induced expression of chaperones, including *HSP70* [112, 113]. In a phase I study, AT13387 was tolerable in patients with advanced solid tumors and had acceptable safety profile. Cardiotoxicity and unfavorable hepatotoxicity observed for ansamycin HSP90 inhibitors were not observed in this study [114]. Importantly, AT13387 was shown to cross the blood–brain barrier [115]. AT13387 is currently evaluated in combination with dabrafenib and trametinib in a phase I clinical trial (Table 1).

**Table 1:** Active clinical trials evaluating the efficacy of HSP90 inhibitors in patients either with melanoma or other malignancies. HSP90 inhibitors that have shown anti-melanoma activity in preclinical studies were included. Data were extracted from https://clinicaltrials.gov

HSP90 inhibitor	Additional drugs	Major inclusion criteria	Phase	Identifier
On-going clinical trials evaluating the efficacy of HSP90 inhibitors in melanoma patients				
XL888	Vemurafenib	BRAF^V600E/K^ mutation; AJCC stage IIIB, IIIC, IV; unresectable	1	NCT01657591
XL888	Vemurafenib + cobimetinib	BRAF^V600^ mutation; unresectable AJCC stage IV, IIIB or IIIC	1	NCT02721459
AT13387	Dabrafenib + trametinib	BRAF^V600E/K^ mutation; metastatic or unresectable	1	NCT02097225
Other active clinical trials on HSP90 inhibitors with known anti-melanoma properties				
AT13387	–	different lymphomas	2	NCT02572453
AT13387	Paclitaxel	Breast cancer triple-negative breast carcinoma	1	NCT02474173
AT13387	AT7519 M	Solid tumors	1	NCT02503709
AT13387	Olaparib	Unresectable solid tumors fallopian tube/ovarian carcinoma triple-negative breast carcinoma	1	NCT02898207
AT13387	Cisplatin	Squamous cell carcinoma	1	NCT02381535
AT13387	Erlotinib	Lung non-small cell carcinoma	1/2	NCT02535338
ganetespib	Crizotinib	Lung cancer	1	NCT01579994
ganetespib	–	Lung cancer	1/2	NCT01590160
ganetespib	Niraparib carboplatin	Fallopian tube/ovarian carcinoma primary peritoneal carcinoma	2	NCT03783949
ganetespib	–	Breast cancer	2	NCT01042379
NVP-AUY922	–	Gastrointestinal stromal tumor	2	NCT01389583
NVP-AUY922	Alpelisib or capmatinib or ceritinib or binimetinib	Adenocarcinoma lung cancer squamous cell lung carcinoma	2	NCT02276027
PU-H71	–	Non-Hodgkin’s lymphoma myeloma solid malignancy	1	NCT01269593
PU-H71	Ruxolitinib	Myelofibrosis	1	NCT03373877
PU-H71	–	Metastatic solid tumor lymphoma	1	NCT01393509
PU-H71	Nab-paclitaxel	Metastatic breast cancer	1	NCT03166085
PU-H71	–	Myelofibrosis	1	NCT03935555
XL888	Pembrolizumab	Colorectal and pancreatic cancer	1	NCT03095781

#### NVP-AUY922 (luminespib)

NVP-AUY922 is a resorcinol isoxazole amide compound with a high affinity to HSP90 [116]. NVP-AUY922 affected proliferation and inhibited colony-forming capacity of melanoma cells. NVP-AUY922 decreased protein level of cyclin D1, and decreased activity of ERK1/2 and NF-κB signaling pathway [117]. In addition to inducing apoptosis, NVP-AUY922 elevated LC3II/LC3I (microtubule-associated proteins 1A/1B light chain 3B) ratio in a time-dependent manner indicating activation of autophagy [117]. Conflicting results of NVP-AUY922 activity on melanoma tumor growth and metastasis were published [116, 117]. NVP-AUY922 induced expression of *HSP70*, *GRP78* and *DDIT3* (DNA damage-inducible transcript 3) encoding CHOP, thereby increasing endoplasmic reticulum stress and activating unfolded protein response in melanoma cells [117]. Co-treatment with PFT-μ (2-phenylethynesulphonamide), which acted as a dual inhibitor of HSP70 and autophagy, showed a synergistic anti-melanoma activity both in vitro and in vivo probably by deregulating redox balance [117].

#### Ganetespib

Ganetespib and its prodrug, STA-1474, are water soluble compounds that bind to N-terminal domain of HSP90, and exert anti-cancer activity [118–120]. Ganetespib destabilized MAPK signaling in melanoma cells by diminution of HSP90 client proteins including CRAF and BRAF^V600E^ leading to attenuation of MEK1/2 and ERK1/2 activity in a concentration-dependent manner, while not affecting BRAF protein level in wild-type *BRAF* melanoma cells and melanocytes [121, 122]. In addition, ganetespib reduced expression of *EGFR*, *IGF1R* (insulin-like growth factor 1 receptor) and *MET*, inhibited AKT activity [121, 123], and upregulated HSP70 [123]. Ganetespib induced melanoma cell cycle arrest in G2, G1 and G2/M phases in a cell line-dependent manner [121]. This was associated with reduction of *CDK1* (cyclin-dependent kinase 1) [121, 123], *CDK2* (cyclin-dependent kinase 2) and *CDK4* expression, and diverse alterations in protein levels of p27^Kip1^ (cyclin-dependent kinase inhibitor 1B), p21^Cip1^ (CDK-interacting protein 1) and cyclins [121]. In addition to cytostatic effect, ganetespib activated caspase-3 and caspase-7 leading to apoptosis in melanoma cells [121, 122]. Ganetespib-induced apoptosis was associated with decrease in protein levels of anti-apoptotic proteins including survivin, BCL-2, BCL-X_L_ (B-cell lymphoma-extra-large) and MCL-1 (myeloid cell leukemia 1), although elevated levels of these proteins were reported in certain cell lines exposed to ganetespib [121]. Ganetespib inhibited tumor growth in mice xenografts [122], significantly potentiated the tumor growth inhibitory effect of BRAF and MEK inhibitors, and overcame mechanisms of primary and acquired resistance of melanoma cells to BRAF inhibitors [121, 122]. Ganetespib exerted similar activity in melanoma cells of different genetic subtypes including those harboring both *BRAF* and *NRAS* as wild-types [121]. More recently, ganetespib was demonstrated to potentiate anti-tumor effect of immunotherapy as it sensitized melanoma cells to T-cell-mediated killing by upregulating interferon response genes, *IFIT1*, *IFIT2*, *IFIT3* (interferon-induced protein with tetratricopeptide repeats 1–3) in vitro and in vivo [124]. In a clinical trial on patients with metastatic uveal melanoma stage IV, progression-free survival was 1.6 months and 1.8 months in patient cohorts that received 200 mg of ganetespib weekly and 150 mg of the drug twice a week, respectively [125]. However, ganetespib was poorly tolerated in uveal melanoma patients as it evoked a significant gastrointestinal toxicity, including increased aspartate aminotransferase and alanine aminotransferase activity, nausea, vomiting and diarrhea [125].

#### Purine-based inhibitor

PU-H71 is a purine-scaffold inhibitor of HSP90 that exerts a broader accessibility to a greater number of undimerized HSP90 conformations than geldanamycin, and PU-H71 activity is less affected by phosphorylation of HSP90 [126]. PU-H71 exerted higher selectivity in targeting HSP90-oncoprotein complexes than several other N-terminal inhibitors of HSP90 [127]. PU-H71 was shown to induce ER stress and activate UPR pathway involving upregulation of *DDIT3* expression. This was followed by loss of mitochondrial membrane potential and activation of caspase-3, culminating in induction of apoptosis in melanoma cells [128]. Interestingly, these effects were also reported in cancer cell lines of different origin, but not in normal fibroblasts and tissues [128, 129]. Selectivity towards cancer cells was also observed for PU-H71-dependent radiosensitization [130]. More recently, a first-in-human study revealed that PU-H71 was well tolerated in patients with refractory solid tumors, and exerted no dose-limiting toxicity with predominantly grade 1 adverse effects [131].

#### Non-resorcinol pyrazole inhibitors

SNX-2112 is an N-terminal domain binding HSP90 inhibitor containing the 2-aminobenzamide scaffold [132]. SNX-2112 more potently inhibited melanoma cell proliferation than 17-AAG [133, 134], and arrested cells in G0/G1 phase of cell cycle in a dose-dependent manner [133]. SNX-2112 induced a time-dependent degradation of HSP90 client proteins crucial for melanoma cell maintenance including AKT, IKKα, BRAF and glycogen synthase kinase 3 beta (GSK-3β) [133]. SNX-2112 also induced apoptosis in melanoma cells [132, 134], which was associated with an activation of caspase-3, caspase-7 and caspase-8, and PARP cleavage. SNX-2112 induced a time-dependent release of cytochrome c from mitochondria and upregulated pro-apoptotic protein BIM, simultaneously leading to down-regulation of BCL-2, BCL-X_L_ and XIAP (X-linked inhibitor of apoptosis protein). In addition, SNX-2112 inhibited AKT/mTOR/p70^S6K^ (ribosomal protein S6 kinase) pathway to induce autophagy [134]. Two other SNX-2112-related agents were tested in melanoma. SNX-5422, a SNX-2112 prodrug, is rapidly metabolized to SNX-2112 by enzymatic hydrolysis, and exerted promising activity in phase I trials involving patients with solid tumors including melanoma [135, 136]. Most adverse effects were grade 1 or 2 in severity, although few events of grade 3 such as diarrhea, non-septic arthritis and thrombocytopenia were also reported [135, 136]. In SNX-7081, another SNX-2112 derivative, a side chain indazole was replaced with indole. SNX-7081 inhibited cancer cell proliferation more efficiently than its parent drug. Both compounds were highly selective towards cancer cells [132].

#### NVP-BEP800

NVP-BEP800 is a fully synthetic N-terminal inhibitor of HSP90 exerting activity against a number of cancer cell lines at nanomolar concentrations. NVP-BEP800 induced degradation of several melanoma oncoproteins including ERBB2, CRAF, BRAF^V600E^ and AKT [137]. NVP-BEP800 exhibited good bioavailability after oral administration. Pharmacokinetic analysis revealed its short half-life of less than 2 h in plasma, and selective retention in tumor cells with half-life of more than 16 h. Notably, no hepatotoxicity was observed in the preclinical studies [137].

#### XL888

XL888 is an orally bioavailable inhibitor of HSP90 exerting selectivity for this chaperone protein over almost 30 kinases [138]. A high-throughput analysis of XL888-mediated perturbations in cell cycle distribution revealed that XL888 activity might depend on the mutation status of driver oncogenes. XL888 induced G2/M phase accumulation of melanoma cells harboring unmutated *BRAF*, *RAS* and *EGFR*, and homozygous P72R variant of p53 [139]. In turn, the presence of a homozygous BRAF^V600E^ variant was predominantly associated with accumulation of XL888-treated melanoma cells in G1 phase of cell cycle [139]. Cytostatic effect of XL888 activity was accompanied with diminution of cell cycle-related protein levels including WEE1 (WEE1 G2 checkpoint kinase), CHK1 (checkpoint kinase 1), CDK1 and CDK4 [138]. In addition, XL888 diminished protein levels of ARAF (ARAF proto-oncogene, serine/threonine kinase) and CRAF leading to attenuation of ERK1/2 activity, and decreased activity of AKT and S6 kinases [138]. XL888 upregulated *BIM* and *BAX* (BCL-2 associated X protein) expression while decreasing protein level of MCL-1 that resulted in apoptosis induction in *NRAS*-mutant melanoma cells. *MCL1* overexpression, however, prevented from XL888-induced apoptosis [138]. Notably, XL888 efficiently exerted similar effects in three-dimensional spheroid cultures, and in a mouse xenograft model of melanoma with milder effect on the activity of MAPK signaling pathway but retaining its inhibitory potential on *CDK4* and *WEE1* expression, and activity of AKT and S6 [138]. In a panel of *NRAS*-mutated melanoma cell lines, XL888 caused degradation of IGF-1Rβ, PDGFR-β, c-MET and VEGFR1 (vascular endothelial growth factor receptor (1), although it surprisingly increased VEGFR2 (vascular endothelial growth factor receptor (2) level in one cell line [140]. XL888 inhibited cell proliferation also in melanoma cells with intrinsic and acquired resistance to BRAF inhibitors that were dependent on different mechanisms including either overexpression of cyclin D1, PDGFR-β or COT, or *NRAS* mutation [141]. XL888 efficiently reduced levels of ARAF, CRAF and cyclin D1, and inhibited AKT, ERK1/2 and S6 activity in resistant melanoma cells. XL888 restored nuclear localization of FOXO3a (forkhead box O3) that was followed by upregulation of *BIM* expression and diminution of MCL-1 level in vemurafenib-resistant cell lines leading to cleavage of caspase-3 and loss of mitochondrial membrane potential [141]. Interestingly, XL888 arrested vemurafenib-sensitive melanoma cells in G1 phase of cell cycle, but G2/M cell accumulation was predominantly reported in matched vemurafenib-resistant cells exposed to XL888 [141]. XL888 decreased number of non-melanoma skin lesions developed as a result of paradoxical MAPK pathway activation in patients treated with vemurafenib. Similarly, XL888 suppressed ERK1/2 activity in *NRAS*-mutant melanoma cell lines exposed to vemurafenib, and this effect was associated with down-regulation of CRAF [142]. In addition, XL888-mediated inhibition of HSP90 was accompanied with induction of a compensatory mechanism involving *HSP70* upregulation as shown in both in vitro and in vivo models of melanoma. HSP70 induction was similarly observed in drug-naïve and resistant melanoma cells [138, 140, 141]. In a clinical study, XL888 in combination with vemurafenib displayed a tolerable side-effect profile and promising activity in melanoma patients with BRAF^V600E^-mutant tumors. Objective responses were reported in 15/20 patients, including 3 complete and 12 partial responses. The most common adverse effects of grade 3 and 4 were rash and diarrhea, and cutaneous squamous cell carcinomas was developed in 14% of patients [143]. XL888 resistance mechanism involving CDK2 was identified in melanoma cells, and *CDK2* expression was dependent on MITF (microphthalmia-associated transcription factor) [144]. XL888 is currently evaluated in phase I clinical trials (Table 1).

### Middle domain inhibitors

#### Sansalvamide A and sansalvamide A derivatives

Sansalvamide A (San A) is a cyclic pentapeptide isolated from the marine fungi *Fusarium sp.* [80, 145]. Sansalvamide A binds to N-terminal fragment of the middle domain of HSP90, and exerts the ability to allosterically disrupt the interactions of C-terminal binding co-chaperones and client proteins [80]. Interestingly, Di-Sansalvamide A (Di-San A), a dimerized derivative of San A, was found to bind C-middle domain of HSP90 suggesting that Di-San A physically prevents from binding of C-terminal binding clients [80]. Three San A-derived compounds, H-10, H-15 and LY-15 were investigated as potential HSP90 inhibitors in melanoma cells. These agents inhibited proliferation in melanoma cell lines in a concentration- and time-dependent manner [146–148]. Additionally, LY-15 and H-10 induced mitochondrial pathway of apoptosis associated with activation of caspase-3 and caspase-9, but not caspase-8 [147, 148]. LY-15 increased BAX and diminished BCL-2 protein levels, and inhibited cell migration [148]. H-15 increased melanin production and upregulated *TYR* (tyrosinase) expression suggesting that H-15 was capable of inducing differentiation in melanoma cells [146].

### C-Terminal domain inhibitors

#### Novobiocin and novobiocin derivative

Novobiocin is an aminocoumarin antibiotic that showed a dose-dependent anti-proliferative effect in melanoma cells, and increased activity of NADPH: cytochrome c reductase and γ-glutamyltranspeptidase [149, 150]. C-terminal domain of HSP90 was identified as a novobiocin-HSP90 interaction surface [151, 152]. Novobiocin induced degradation of HSP90 client proteins including ERBB2, CRAF, mutated p53 and SRC (proto-oncogene tyrosine-protein kinase SRC), although only when used at relatively high concentrations [152]. To improve activity, novobiocin analogues were synthesized with several structural modifications. In one of them, KU135, the coumarin core was modified, and the noviose sugar was replaced with a methylated phenol that can participate in hydrogen bonding [153]. KU135 arrested melanoma cells in the G2/M phase of cell cycle by increasing the phosphorylation of CDC25C (cell division cycle 25C) at Ser216 and diminishing cyclin B level in contrast to novobiocin that did not influence the level of both proteins [154]. Moreover, KU135 reduced melanoma cell viability more potently than novobiocin and N-terminal inhibitor, 17-AAG. KU135-induced apoptosis was associated with dissipation of mitochondrial membrane potential that led to the release of cytochrome c, activation of caspase-8, caspase-9 and caspase-3, and PARP cleavage [154]. Interestingly, KU135 induced AKT phosphorylation after short incubation, but AKT activity was attenuated after 48 h. In addition, KU135 inhibited ERK1/2 activity that might be related to the reduction of HSP90 client proteins, BRAF and CRAF [154]. Unlike N-terminal inhibitors, KU135 did not affect *HSP27* (heat shock protein 27), *HSP70* and *GRP94* expression, and activity of HSF-1 [154].

## Conclusions

Cancer cells are under constant stress due to the presence of mutant proteins and rapid cell proliferation that affects the control of proteostasis, and in turn elevates cell dependence on HSP90. HSP90 is a promising therapeutic target in melanoma as HSP90 clients have been identified among melanoma-associated oncoproteins involved in determination of cell phenotype and response to drugs. Several HSP90 inhibitors exerting anti-melanoma activity in pre-clinical in vitro and in vivo studies are currently evaluated in clinical trials (Table 1). Further research is, however, necessary to more precisely define unique isoforms or conformational preferences for particular HSP90 inhibitors. Limited reports addressing this issue suggest that substantial differences in both client and drug preferences can exist for HSP90α and HSP90β isoforms, and indicate that geldanamycin and ganetespib bind to HSP90β with greater affinity than to HSP90α [155]. Recent advance showing that activity of HSP90 inhibitors can be monitored by using [^11^C]NMS-E973 as a PET tracer to both quantify HSP90 level in vivo and to determine HSP90 occupancy after treatment with HSP90 inhibitors [123] has provided a tool for in-depth discoveries.

## Abbreviations

17-AAG: 17-*N*-Allylamino-17-demethoxygeldanamycin (tanespimycin)
17-AG: 17-Aminogeldanamycin (IPI-493)
17-DMAG: 17-Dimethylaminoethylamino-17-demethoxygeldanamycin (alvespimycin)
BAX: BCL-2 associated X protein
BCL-2: B-Cell CLL/lymphoma 2
BCL-X_L_: B-Cell lymphoma-extra large
BIM: BCL-2-interacting mediator of cell death
BRAF: B-Raf proto-oncogene serine/threonine kinase
c-MET: Hepatocyte growth factor receptor
CDC37: Cell division cycle 37
CDK1/2/4: Cyclin-dependent kinase 1/2/4
COT: Cancer Osaka thyroid oncogene
CRAF: RAF-1 proto-oncogene, serine/threonine kinase
DDIT3: DNA damage-inducible transcript 3
EGFR: Epidermal growth factor receptor
EIF2α: Eukaryotic initiation factor 2
ER: Endoplasmic reticulum
ERK1/2: Extracellular signal-regulated kinase 1/2
GRP78/BiP: Glucose-regulated protein 78/binding immunoglobulin protein
GRP94: Glucose-regulated protein 94
HIF-1α: Hypoxia-inducible factor 1
HSF-1: Heat shock factor 1
HSP70/90: Heat shock protein 70/90
IRE1α: Inositol-requiring enzyme 1 alpha
MCL-1: Myeloid cell leukemia-1
MEK1/2: Mitogen-activated protein kinase 1/2
mTOR: Mechanistic target of rapamycin kinase
NF-κB: Nuclear factor-kappa B
NRAS: Neuroblastoma RAS viral oncogene homolog
NQO1: NAD(P)H:quinone oxidoreductase 1
PARP1: Poly(ADP-ribose) polymerase 1
PD-1: Programmed cell death protein-1
PI3 K: Phosphatidylinositol 3-kinase
UPR: Unfolded protein response
WNT: Wingless-type
